# Supplementary material for: Rapid Identification of Benign Gallbladder Diseases Using Serum Surface-Enhanced Raman Spectroscopy Combined with Multivariate Statistical Analysis
Source: Diagnostics (Basel). 2023 Feb 8;13(4):619. doi: 10.3390/diagnostics13040619 (PMC9955438; doi:10.3390/diagnostics13040619)
Supplement: Supplementary file 1 [file diagnostics-13-00619-s001.zip › diagnostics-2131894-supplementary.pdf]

Supplementary material:

Rapid identification of benign gallbladder diseases using serum surface-enhanced Raman spectroscopy combined with multivariate statistical analysis

Wubulitalifu Dawuti[a,b,1], Jingrui Dou[a,b,1], Jintian Li[a,b], Hui Liu[a], Hui Zhao[c], Li Sun[a], Jin Chu[a], Renyong Lin[a*], Guodong Lü[a*]

[a] State Key Laboratory of Pathogenesis, Prevention, and Treatment of Central Asian High Incidence Diseases, Clinical Medical Research Institute, The First Affiliated Hospital of Xinjiang Medical University, Urumqi, Xinjiang, China
[b] School of Public Health, Xinjiang Medical University, Urumqi, Xinjiang, China
[c] Department of Clinical Laboratory, The First Affiliated Hospital of Xinjiang Medical University, Urumqi, China
* Corresponding author at: State Key Laboratory of Pathogenesis, Prevention, and Treatment of Central Asian High Incidence Diseases, Clinical Medical Research Institute, The First Affiliated Hospital of Xinjiang Medical University, No. 137 Liyushan South Road, Urumqi, 830054, China.
E-mail addresses: lgd_xj@qq.com (G. Lü), renyonglin@xjmu.edu.cn (R. Lin).
[1] Contribution equally.

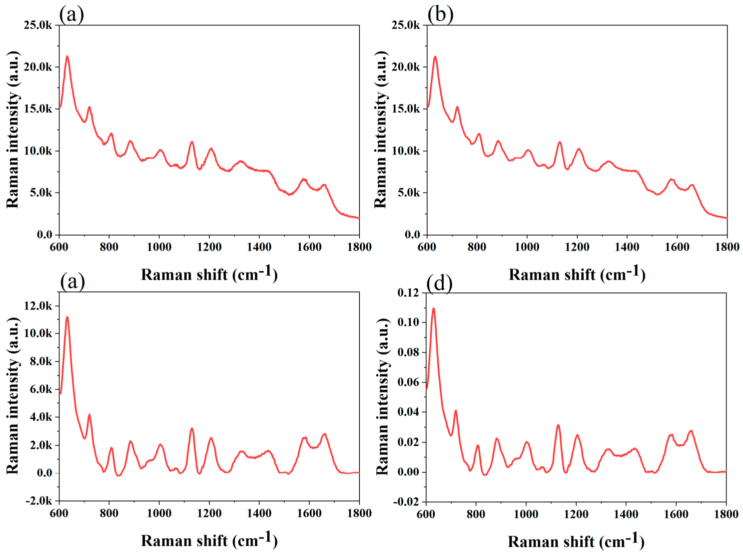

Figure.S1. SERS spectral preprocessing results of a selected Healthy sample; (a) Raw spectrum;(b) The spectrum of the raw spectrum after denoising; (c) Baseline corrected spectrum; (d) Normalized spectrum.

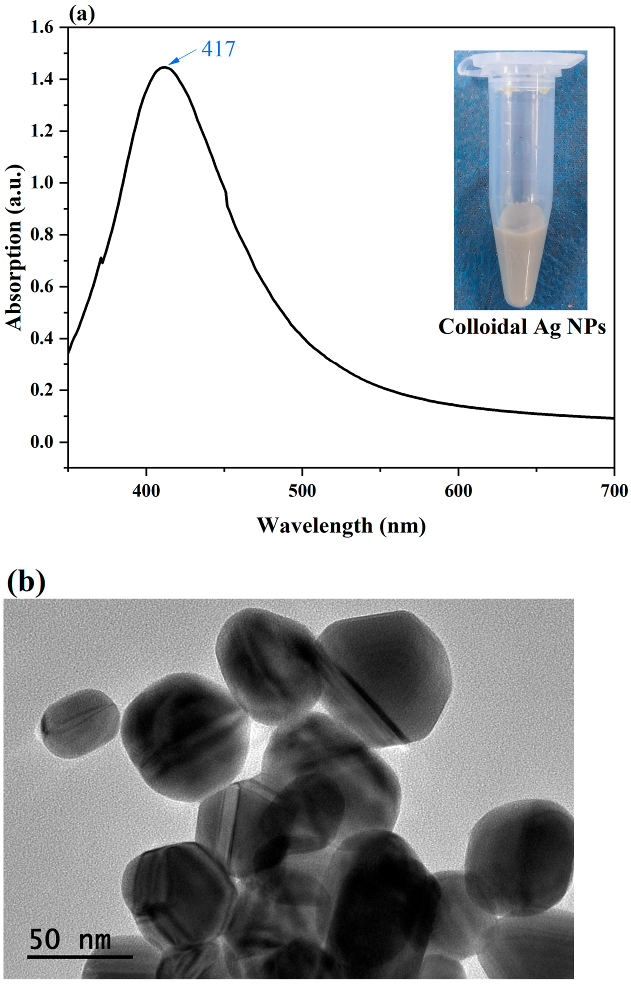

Figure.S2. (a) The UV/visible absorption spectrum of the Ag colloid. The absorption maximum is located at 417 nm; (b) Transmission electron microscopy (TEM) image of Ag nanoparticles (NPs).

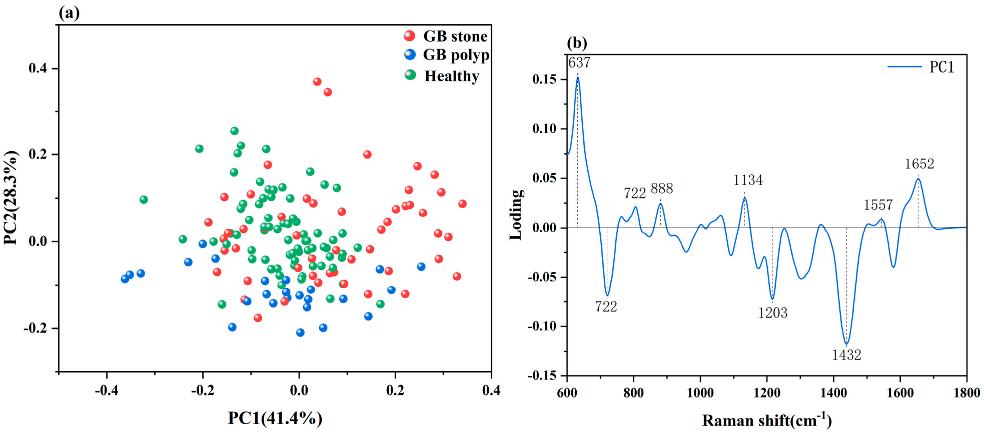

Figure.S3. (a) Principal component score plots of the SERS spectra based on PCA-LDA; (b) Loading plot for the first PC. The first PC accounting for 41.4% of the total variance.

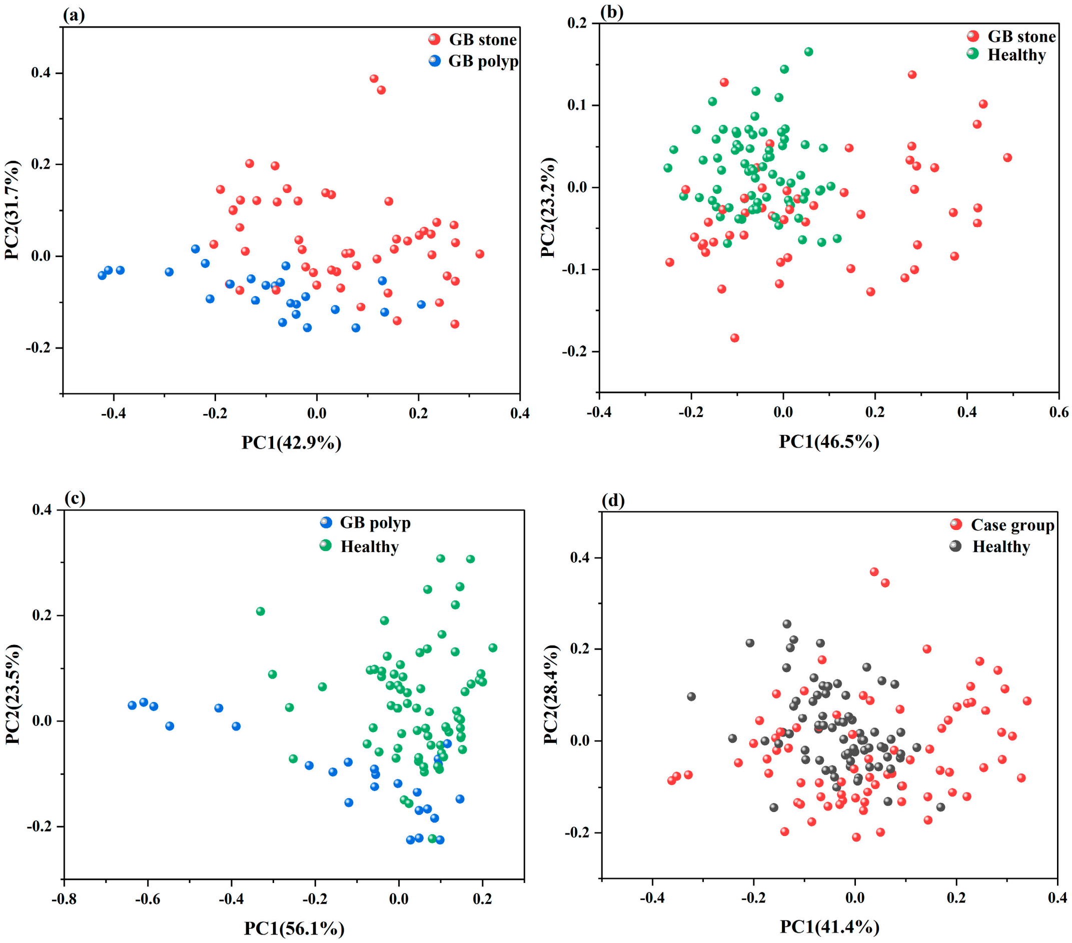

Figure.S4. Principal component score plots of the SERS spectra based on PCA-LDA. (a) Score plot for GB stone, and GB polyp serum samples. (b) Score plot for Healthy, and GB stone serum samples. (c) Score plot for Healthy, and GB polyp serum samples. (d) Score plot for Healthy, and Case group serum samples.
